# Predicting college students’ exercise dependence: a machine learning approach

**DOI:** 10.3389/fpsyg.2026.1743725

**Published:** 2026-01-29

**Authors:** Yihang Deng, Wei Lan, Mingda Si, Yi Lin Ren

**Affiliations:** 1 Department of Physical Education, Neijiang Normal University, Neijiang, China; 2 Embodied Media Laboratory, Graduate School of Media Design, Keio University, Hiyoshi Campus, Yokohama, Japan; 3 National Institute of Education, Nanyang Technological University, Singapore, Singapore; 4 Zhuhai Research Center for Women and Children’s Sports Culture, College of Sports, Jinan University Zhuhai Campus, Zhuhai, Guangdong, China

**Keywords:** college student, ensemble learning (EN), exercise dependence behavior, machine learning, risk prediction

## Abstract

Exercise dependence behavior among college students is a critical issue in sports psychology that deserve closer examination, and artificial intelligence offer a useful ways to explore its mechanisms and predicting associated risks. In this study, data were collected from 2,745 college students using three standardized questionnaires, covering (i) exercise dependence behavior, (ii) psychological characteristics (e.g., exercise identity, weight biases), and (iii) basic demographic information. We used four widely used machine learning algorithms: logistic regression, random forest, extreme gradient boosting (XGBoost), and multilayer perceptron, and their outputs were further integrated through an ensemble learning techniques to further enhance the robustness and predictive power of the models. The stacking ensemble model achieved a mean AUC of 0.96 in identifying exercise dependence risk among college students, demonstrating that integrating multiple machine learning approaches can yield robust and highly accurate risk prediction in this setting. Among the variables, the most influential predictors of exercise dependence behavior included prolonging exercise to obtain the desired effect, allocating most leisure time on exercise, experiencing difficulty in reducing exercise frequency, and actual exercise time longer than originally planned. These findings uncovers the key psychological and behavioral mechanisms underlying in exercise dependence among college students and show that artificial intelligence methods can be effectively applied to support risk monitoring in sport and psychological health contexts.

## Highlights

- This study applies advanced machine learning algorithms to predict exercise dependence among Chinese college students.
- Deviation regulation theory and exercise psychology provide the theoretical foundation for identifying key psychological predictors.
- Exercise identity and weight bias are found to be significant psychological variables influencing exercise dependence.
- The use of data-driven methods improves prediction accuracy compared to traditional statistical approaches.
- Findings offer new perspectives for early identification and intervention strategies targeting exercise dependence in university populations.

## Introduction

Regular physical activity is universally recognized as a building block of both physical and mental health, which has consistently been associated with positive health, better performance at school and work place (Calestine et al., 2017; Mahindru et al., 2023; Ruegsegger and Booth, 2018). Yet, as exercise load increases, the beneficial effects often decrease and may even be converted to maladaptive or addictive behavior. A highly relevant behavioral condition in this context is exercise dependence (ED). Exercise dependence (ED) is defined as a dysfunctional behavior pattern that is accompanied by withdrawal-like effects, such as anxiety and depression if the individual has to refrain from exercising, and an uncontrollable desire to increase workout intensity or frequency (de Coverley Veale, 1987). Systematic reviews estimate that approximately 3–7% of college students and regular exercisers are at risk for ED, with prevalence rates notably higher in athletic populations (Lichtenstein et al., 2018).

Importantly, this is a phenomenon that deserves special consideration in the Chinese higher education system given rapid sociocultural changes happening to student lives. Inspired by the nationwide “Healthy China 2030” program, fitness activities participation has increased significantly among Chinese youth (Chen et al., 2019). However, this wave of fitness meets some special stressors: college students in China face fierce academic pressure (commonly known as “involution”) and severe body image anxiety brought by the increasingly popular trend of social media (Wang et al., 2023). There is empirical evidence that these pressures may increase the risk for maladaptive exercise. Data from recent epidemiological surveys have shown that the proportion of Chinese college students with the potential risk for exercise dependence is 6.6 to 10.5% in active subpopulations, which is similar or even higher than the prevalence rates described in some Western data sources (Gong et al., 2023; Zhang, 2025). Despite these alarming trends, exercise dependence is rarely studied in the context of Chinese universities.

Despite several decades of research on exercise dependence (de Coverley Veale, 1987), most empirical studies have focused on competitive athletes and fitness enthusiasts (Szabo and Demetrovics, 2022), and fewer studies have examined college students in a systematic way (Terry et al., 2004). Moreover, much of the literature has been framed through a pathological lens, underscoring symptoms and diagnostic features (Berczik et al., 2012; Hausenblas and Symons Downs, 2002) rather than applying psychological theories to elucidate underlying mechanisms. For instance, prior work has predominantly relied on diagnostic criteria, such as tolerance, withdrawal, and loss of control, to classify high-risk individuals, but has paid comparatively less attention to the motivational and social-cognitive processes that may lead students to develop maladaptive exercise patterns (Griffiths et al., 2005; Szabo et al., 2013). By contrast, comparatively fewer studies have applied psychological theories to explain maladaptive exercise behaviors. One promising framework is deviance regulation theory (DRT), which argues that people regulate their behavior in order to preserve a valued social identity in relation to salient group norms.

According to DRT, people’s behavior is responsive to their estimates of the group norm with which they compare themselves: individuals are motivated to self-enhance when that category good is rare (e.g., exercising when most peers do not exercise) or to avoid attention when it is prevalent but potentially harmful (Blanton et al., 2001; Blanton and Christie, 2003). Within the exercise domain, message framing consistent with DRT has been found to affect intentions and subsequent behavior in reliance on perceived group norms and identity-relevant depictions (Gallagher and Updegraff, 2012). In addition, DRT has been used to explore exercise identity alongside social factors like weight bias in predicting excessive training or muscle building exercise (Palermo et al., 2021).

Drawing from this framework, the present study views each predictor as a theoretically justified proxy for either (a) perceived deviation from salient exercise norms or (b) conflict between one’s exercise identity and broader social expectations. So, for example, it has been shown that stronger exercise identity is associated with greater risk of exercise dependence when athletes perceive their own training volume or intensity exceeds what the general group norm would predict because deviation from the group norm may be a metaphor of why was believed to be a “discipline” self (Hausenblas and Symons Downs, 2002; Miller and Mesagno, 2014). On the other hand, pupils with a less strong exercise identity may not be as proned to risk compulsive behaviors because they are not highly motivated and do not contribute to develop different compulsive patterns in order to maintain an exclusive in-group status via high training commitment.

However, a major drawback of the current exercise dependence field is that much of the research has been conducted using conventional linear statistical methods [i.e., multiple regression, ANOVA, and structural equation modeling (SEM)] (Chang et al., 2019; Lichtenstein et al., 2014a). Although these methods have their strengths, they base on the assumption of linearity and additivity, i.e., that the association between psychological correlates (e.g., identity, anxiety) and exercise dependence is also linear across all participants (Yarkoni and Westfall, 2017). As a consequence, these models can suffer from modeling only part of the non-linear interactions that are known to describe behavioral psychopathology, thus leading to rather modest explained variance and predictive accuracy (Bzdok et al., 2018). For example, the relationship between exercise identity and dependence may not be constant, but rather depend on different levels of social pressure or body dissatisfaction, an exchange that is difficult to account for using traditional linear interaction terms in high-dimensional datasets (Strobl et al., 2009). In the specific exercise dependence literature, prior work has typically modeled a restricted set of predictors using such linear models, yielding only modest explained variance and offering limited insight into how identity-related and contextual factors jointly shape maladaptive exercise patterns.

To address these methodological shortcomings, machine learning (ML) techniques offer a robust alternative. Unlike traditional inference-based statistics, ML prioritizes out-of-sample prediction and can flexibly model high-dimensional, non-linear relationships without strict parametric assumptions (Breiman, 2001; Yarkoni and Westfall, 2017). In psychology research, these methods have already been used in areas such as emotion recognition (Khare et al., 2024), psychopathology (Bartlett et al., 2014; Livieris et al., 2018), and health behaviors (Abdul Rahman et al., 2023; Goh et al., 2022). Specifically, ML offers distinct advantages for this study: (1) the use of cross-validation (splitting data into training, validation, and test sets) minimizes overfitting and provides more generalizable performance estimates (Lu, 2010); (2) ensemble algorithms (e.g., random forest, XGBoost) can aggregate weak learners to significantly enhance classification accuracy over single-model approaches (Chen and Guestrin, 2016); (3) integration with explainable AI tools, such as SHAP (SHapley Additive exPlanations), allows for the transparent identification of variable contributions, overcoming the “black box” criticism of complex models (Lundberg and Lee, 2017); and (4) ML-based models can generate individual risk estimates, facilitating early detection and targeted intervention. Despite these clear advantages, to our knowledge, no study has yet combined DRT with ML techniques to disentangle the complex psychological architecture of exercise dependence among college students.

Grounding in deviance regulation theory (DRT) and by means of machine learning techniques, the present study examines exercise dependence among Chinese college students. Importantly, by positing exercise dependence as a maladaptive mode of social identity regulation, DRT directs us in selecting our features. We chose exercise identity as a core predictor, a dispositional motive to differentiate oneself positively from others (Strachan et al., 2011), and weight bias as an important contextual stressor—the concern about standing apart from group body norms (Puhl and Suh, 2015). And demographic variables (gender, grade, BMI) were not just included only as covariates but correlated with social comparison groups for which individuals’ behaviors are calibrated against (Blanton et al., 2001). By including these variables in machine-learning models, this study seeks to (1) identify essential factors that are primarily related with exercise dependence, (2) examine their relative contributions in predicting exercise dependence, and (3) offer scientific insights for the design of specific prevention and intervention plans.

Building directly on DRT, we formulated the following theory-driven hypotheses: (H1) Machine learning models will show improved predictive performance (higher accuracy and AUC) as compared to traditional logistic regression baselines because they account for non-linear dependencies; and (H2) Consistent with DRT’s focus on identity preservation, exercise identity and weight bias will rise to be the top-ranking predictors that interact such that students with high exercise identity who also experience high weight bias are predicted to have the highest probability of exercise dependence.

## Methods

### Participants

This study was approved by the Academic Ethics Committees of the University (Ethics No. YZUHL2020102), and informed consent was obtained from the students’ respective colleges and administrative units. Undergraduate students were recruited from several colleges, and classes were randomly selected from each of the four-year levels (freshman to senior). Data were collected online using Wenjuanxing (Questionnaire Star), which enabled students to complete the survey on their smartphones. A total of 2,920 questionnaires received, 106 questionnaires with less than 70% completion and 69 questionnaires missing values on key outcome variables were excluded. The final sample consisted of 2,745 students, giving a valid response rate of 94.01%. The average age was 20.37 ± 1.53 years, with males comprising 43.4% of participants. Table 1 summarizes the demographic characteristics (grade, gender, BMI).

**Table 1:** Basic characteristics of the study population.

Demographic variables	General information	Number	Percentage
Gender	Male	1,192	43.4%
	Female	1,553	56.6%
Grade	Freshman	688	25.1%
	Sophomore	675	24.6%
	Junior	819	29.8%
	Senior	563	20.5%
BMI	Thin	436	15.9%
	Normal	1,720	62.7%
	Overweight	412	15.0%
	Obese	177	6.4%

### Measures

#### Outcome variable measurement: Exercise Dependence Scale-Revised

Exercise dependence behavior was assessed using the Chinese version of the Exercise Dependence Scale-Revised (EDS-R) adapted by Yang et al. (2021). The EDS-R includes 21 items across seven dimensions, scored on a 6-point scale (1 = never, 6 = always). Higher scores means more dependence-like exercise behavior. The scale demonstrated excellent internal consistency (Cronbach’s alpha = 0.977). Participants scoring 15 or higher on at least three dimensions were classified as at risk for exercise dependence (ARED). Those not classified as ARED but scoring 7 or higher on three dimensions were categorized as non-dependent symptomatic (NDS). Participants not meeting the criteria for either group were classified as non-dependent.

#### Predictor variable measurement: Exercise Identity Scale

Exercise identity was accessed with the Chinese version of the Exercise Identity Scale (EIS), originally developed by Anderson and Cychosz (1994) and revised by Li et al. (2019). This unidimensional scale consists of 9 items rated on a 7-point scale (1 = strongly disagree, 7 = strongly agree), with higher scores indicating a stronger exercise identity. The Cronbach’s alpha for the EIS in this study was 0.937. From a DRT perspective, exercise identity functions as an internalized standard for “being a disciplined exerciser,” making discrepancies from perceived peer norms particularly salient.

#### Predictor variable measurement: Eating Pathology Symptoms Inventory

Weight bias was measured with a 5-item subscale from the Eating Pathology Symptoms Inventory (ESPI) that measures negative attitudes towards obesity (*α* = 0.89). The ESPI is a 45-item self-report instrument evaluating eating disorder symptoms, with items rated on a 5-point scale (1 = never, 5 = very often), reflecting symptom frequency over the past 4 weeks (Forbush et al., 2013). Because no Chinese version of this subscale was available, we translated and evaluated its psychometric properties. Detailed confirmatory factor analysis (CFA) results supporting its reliability and validity in this sample are reported in the Results section. Within DRT, internalized weight bias is interpreted as a contextual pressure to avoid the stigmatized identity of being “unfit,” potentially motivating excessive exercise among students striving to conform to perceived body norms.

#### Demographic information questionnaire

The self-developed demographic questionnaire included three sections: gender, year of study (freshman to senior), height, and weight, BMI was calculated from height and weight.

#### Theoretical mapping of predictors to DRT

In line with DRT, all predictors were coded as indicators of either (a) perceived deviations from salient exercise norms (for example, training volume and dependence symptoms) or (b) discrepancies between the exercise self-concept and social norms regarding what it means to fit in with a given social group (internalized weight bias, demographic reference groups). Greater exercise identity and stronger weight bias would be expected to predict dependence risk, especially with high levels of behavioral engagement (e.g., long duration, frequent engagement).

### Data analysis

#### Machine learning model construction

Descriptive statistics were conducted using SPSS 27.0 to summarize sample characteristics. Subsequent machine learning modeling was implemented in Python (version 3.9, using Scikit-learn and XGBoost libraries). Following the best-practice guidelines for prediction modeling as outlined in the TRIPOD statement (Collins et al., 2015), we adopted a systematic workflow to ensure model transparency and reproducibility. Given that the relations between the predictors and exercise dependence were likely to be non-linear, we trained four classifiers with four different modeling assumptions: multinomial logistic regression (MLR), random forest (RF), eXtreme gradient boosting (XGBoost), and multilayer perceptron (MLP). These algorithms were selected to capture both simpler and more complex patterns in the data, so that key factors related to exercise dependence in college students could be captured more fully. A stacking ensemble was finally used to combine the four base models in order to obtain a more stable and accurate classifier.

#### Four machine learning algorithms and procedures

The modeling steps comprised: (1) Data preprocessing: Continuous variables were *Z*-score standardized and categorical variables were dummy coded. (2) Data splitting: The data were split randomly into a training set (64%), a test set (16%), and a hold-out set (20%). This allocation effectively retains 80% of the data for model development (encompassing training and internal tuning) while reserving a strictly independent 20% hold-out sample to provide an unbiased estimate of generalization performance (Varma and Simon, 2006; Yarkoni and Westfall, 2017). (3) Model design and training: Five machine-learning algorithms are adopted, including logistic regression (LR), random forests (RF), XGBoosting (XGB), a fully-connected neural network model (MLP) and one stacking ensemble. Hyperparameters settings were chosen in a way to guarantee reproducibility and convergence:

- LR: With multinomial strategy (multi_class = “multinomial”) and maximum number of iterations 1,000 (max_iter = 1,000) to ensure convergence of the solver.
- RF: Used the default parameters and set the random seeds to get deterministic results.
- XGB: Set up for multi-class log loss optimization (eval_metric = “mlogloss”).
- MLP: A two hidden layers model with 64 and 32 neurons (hidden_layer_sizes = (64, 32)), trained at most for 1,000 iterations.
- Stacking classifier: Combine the above four models (LR, RF, XGB and MLP) as base models and a logistic regression model (same hyperparameters as a single model of LR) is used as meta-model.

(4) Model assessment: Model performance was measured on test set using accuracy, precision, recall and *F*_1_-score. (5) Robustness validation: For alleviation of sampling bias, the training-test process was repeated 100 times with random splits. The last model of all models was validated in the holdout set to evaluate generalization.

#### Evaluation metrics

To strictly and comprehensively evaluate the performance of the machine learning models, we employed several key metrics: area under the curve (AUC), accuracy, precision, recall, and average precision (AP). Additionally, model stability was assessed using the standard deviation (SD) of accuracy scores across cross-validation folds.

*ROC curve and AUC*: The area under the curve (AUC) is calculated as the integral of the receiver operating characteristic (ROC) curve, which plots the true positive rate (TPR) against the false positive rate (FPR). The AUC is defined as:


$$\mathrm{AUC}=\int_{0}^{1}\mathrm{TPR}(\mathrm{FPR})d(\mathrm{FPR})$$

where TPR = $\;\frac{\mathrm{TP}}{\mathrm{TP}+\mathrm{FN}}(\text{sensitivity}),$ FPR = $\;\frac{\mathrm{FP}}{\mathrm{FP}+\mathrm{TN}}$ (1-sensitivity)

*Confusion matrix-based metrics*: The calculations for precision, recall, and accuracy are derived from the confusion matrix, which consists of true positives (TR), false positives (FP), true negatives (TN), and false negatives (FN).

*Accuracy*: Accuracy represents the overall correctness of the model and is calculated as the ratio of correctly predicted observations to the total observations:


$$\text{Accuracy}=\frac{\left(\mathrm{TP}+\mathrm{TN}\right)}{\left(\mathrm{TP}+\mathrm{TN}+\mathrm{FP}+\mathrm{FN}\right)}$$

*Precision*: Precision measures the accuracy of positive predictions. It is defined as the ratio of correctly predicted positive observations to the total predicted positive observations:


$$\text{Precision}=\frac{\mathrm{TP}}{\left(\mathrm{TP}+\mathrm{FP}\right)}$$

*Recall*: Recall (also known as sensitivity) measures the ability of the model to identify all relevant cases (positive samples). It is the ratio of correctly predicted positive observations to all observations in the actual class:


$$\text{Recall}=\frac{\mathrm{TP}}{\left(\mathrm{TP}+\mathrm{FN}\right)}$$

*F_1_-score*: *F*_1_-score is employed as a comprehensive metric to evaluate the overall performance of the model. Defined as the harmonic mean of precision and recall, the *F*_1_-score provides a balanced assessment by taking both false positives and false negatives into account. It is particularly useful for comparing classifiers, as it penalizes extreme values in either precision or recall. The formula is calculated as follows:


$$F_{1}-\text{score}=2\times \frac{\text{Precision}+\text{Recall}}{\text{Precision}\times \text{Recall}}$$

*Average precision*: Average precision (AP) summarizes the precision-recall curve into a single value representing the average of all precision values calculated at each threshold level. It is calculated as the weighted mean of precisions achieved at each threshold, with the increase in recall from the previous threshold used as the weight:


$$\mathrm{AP}=\sum_{0}(R_{n}-R_{n-1})P_{n}$$

where $R_{n}$ and $R_{n-1}$ are the precision and recall at the *n*-th threshold, respectively.

*Model stability*: To evaluate model stability and robustness across the cross-validation folds, we utilized the standard deviation (SD) of the accuracy scores. A lower SD indicates higher stability: $\mathrm{SD}=\sqrt{\frac{1}{k-1}\sum_{i=1}^{k}{\left({\mathrm{ACC}}_{i}-\bar{\mathrm{ACC}}\right)}^{2}}$

where *k* is the number of folds (or repetitions), ${\mathrm{ACC}}_{i}$ is the accuracy of the *i*-th fold, and $\bar{\mathrm{ACC}}$ is the mean accuracy.

#### Integration and evaluation

In the end, predictions of the four base models were stacked in a stacking ensemble where logistic regression acted as meta-learner, performance was checked using five-fold cross-validation. Model performance was described as AUC and SHAP. The combination of these set-up allowed identifying both model accuracy and the relative significance of sensitive parameters.

## Results

### Confirmatory factor analysis

**Figure 1:**
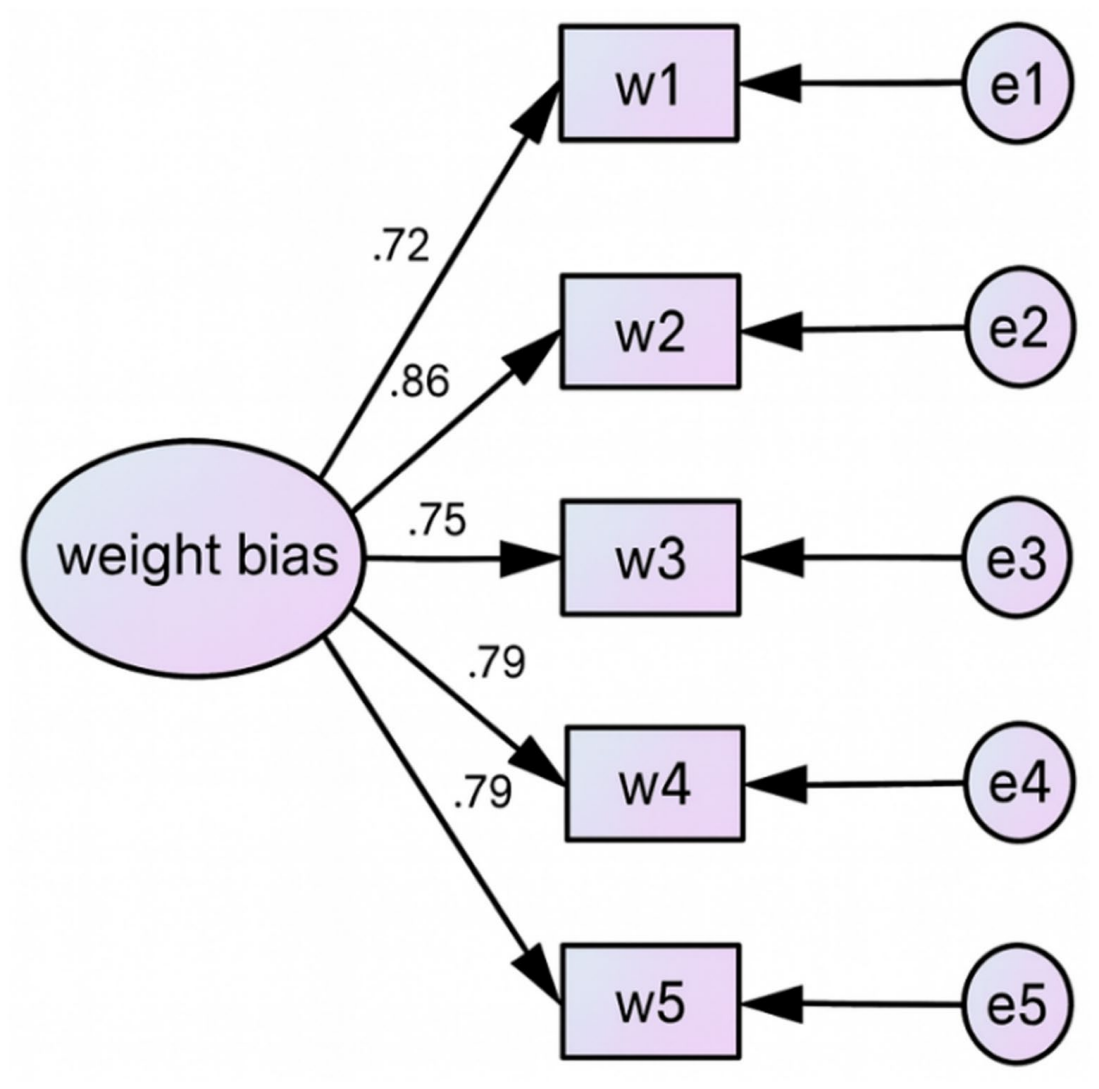
Confirmatory factor analysis of the weight bias scale.

Confirmatory factor analysis (CFA) was conducted via the AMOS 26.0 program to evaluate the structural validity of both the Weight Bias Subscale and Exercise Dependence Scale. The goodness of fit was checked against well-accepted indices and criteria (Hair et al., 2009; Hu and Bentler, 1999). The model demonstrated an excellent fit to the data, with reported indices as follows: *χ*^2^/df = 1.033 (suggesting a good fit as it is <3.0; McIver and Carmines, 1981), RMSEA = 0.023 (<0.06; Hu and Bentler, 1999), CFI = 0.983 (>0.90; Bentler, 1990), IFI = 0.996, TLI = 0.978 (>0.90; Tucker and Lewis, 1973), and PNFI = 0.654 (>0.50; Mulaik et al., 1989). This suggests that the measurement model was statistically sound and suitable for further machine learning analyses (see Figure 1 and Table 2).

**Table 2:** Model fit indices for the weight bias scale.

Index	Absolute fit index		Incremental fit index			Parsimony fit index
	*χ*^2^/df ⬇	RMSEA ⬇	IFI ⬆	TLI ⬆	CFI ⬆	PNFI ⬆
Criteria	<3	<0.08	>0.9	>0.9	>0.9	>0.5
Fit effect	1.033	0.023	0.996	0.978	0.983	0.654

*χ*^2^/df, chi-square to degrees of freedom ratio; RMSEA, root mean square error of approximation; IFI, incremental fit index; TLI, Tucker–Lewis index; CFI, comparative fit index; PNFI, parsimony-adjusted normed fit index.

### Descriptive statistics

#### Participant grouping by exercise dependence

**Figure 2:**
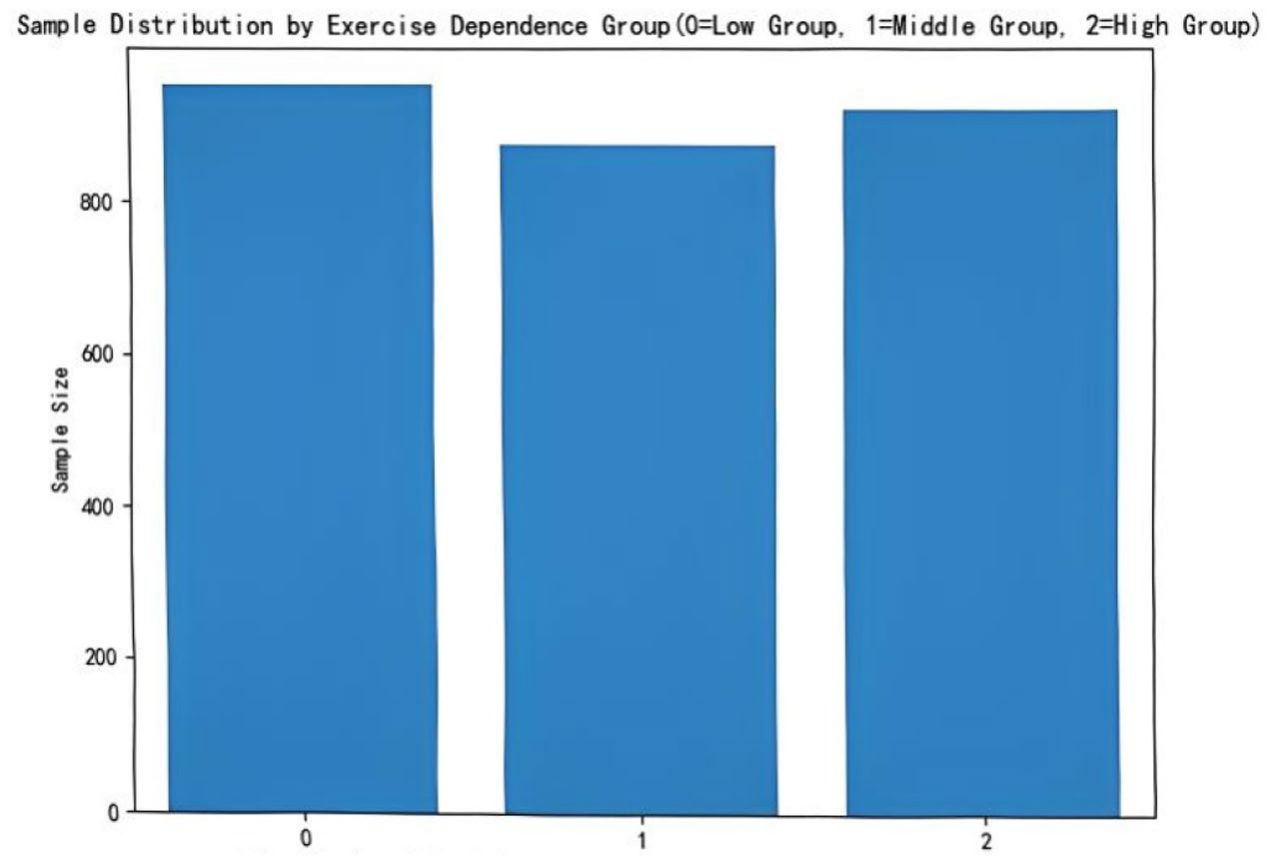
Distribution of participants by exercise dependence total score groups.

For machine learning classification purposes, participants (*N* = 2,745) were categorized into three levels based on their Exercise Dependence Scale scores: no dependence symptoms (No-DS; *n* = 952, 34.68%), non-dependence symptoms (NDS; *n* = 874, 31.84%), and at risk for exercise dependence (ARED; *n* = 919, 33.48%). However, a chi-square test showed that the proportions of participants across these categories did not differ significantly (*χ*^2^ = 3.36, *p* > 0.05). Therefore, the three-level structure was retained for the machine learning analyses, whereas descriptive and inferential statistics were reported for the total sample rather than by group (see Figure 2).

#### Differences in exercise dependence behavior across demographic factors

To explore whether exercise dependence varied by gender, year in school and BMI, we ran independent samples t-tests and one-way ANOVAs. As shown in Table 3, male students scored higher than females on exercise identity, weight bias, and exercise dependence (all *p*-values <0.001). For Grade difference, the first year and the second years’ students reported higher scored than students in other grades. For BMI, students in the normal-weight range scored shows higher exercise identity, higher weight bias, and higher exercise dependence than both underweight and overweight groups. In addition, overweight/obese students reported higher exercise dependence than underweight students. Overall, these findings indicate clear demographic differences in factors linked to exercise dependence.

**Table 3:** Difference analysis of college students’ exercise identity, weight bias and exercise dependence behavior.

Group/Indicator	Exercise identity	Weight bias	Exercise dependence
Male (*N* = 1,192)	39.93 ± 12.20	12.96 ± 5.75	59.4 ± 26.34
Female (*N* = 1,553)	34.80 ± 11.08	11.21 ± 4.81	47.31 ± 19.97
*t*	11.362^**^	8.483^**^	13.206^**^
Freshman (*N* = 688)	38.12 ± 10.74	12.72 ± 5.20	54.58 ± 22.53
Sophomore (*N* = 675)	37.95 ± 11.26	12.45 ± 4.87	54.67 ± 24.32
Junior (*N* = 819)	36.02 ± 12.68	11.71 ± 5.57	50.50 ± 23.98
Senior (*N* = 563)	36.06 ± 12.42	10.86 ± 5.35	50.56 ± 23.64
*F*	6.574^**^	15.631^**^	6.895^**^
LSD	1, 2 > 3, 4	1, 2 > 3, 4	1, 2 > 3, 4
Thin (*N* = 436)	34.67 ± 11.08	10.62 ± 4.50	44.32 ± 18.94
Normal (*N* = 1720)	37.70 ± 12.00	12.33 ± 5.49	54.82 ± 24.58
Overweight (*N* = 412)	37.39 ± 11.55	12.15 ± 5.23	52.84 ± 22.94
Obese (*N* = 177)	35.48 ± 12.14	11.42 ± 5.06	50.22 ± 22.99
*F*	8.78^**^	12.96^**^	23.90^**^
LSD	b > a, d; c > a	b > a, d; c > a	b > a, d; c, d > a

^*^p < 0.05 and ^**^p < 0.01; “1” indicates freshman; “2” indicates sophomore; “3” indicates junior; “4” indicates senior; “a” indicates underweight; “b” indicates normal; “c” indicates overweight; “d” indicates obese.

#### Exercise dependence behavior and its associated factors in college students

**Figure 3:**
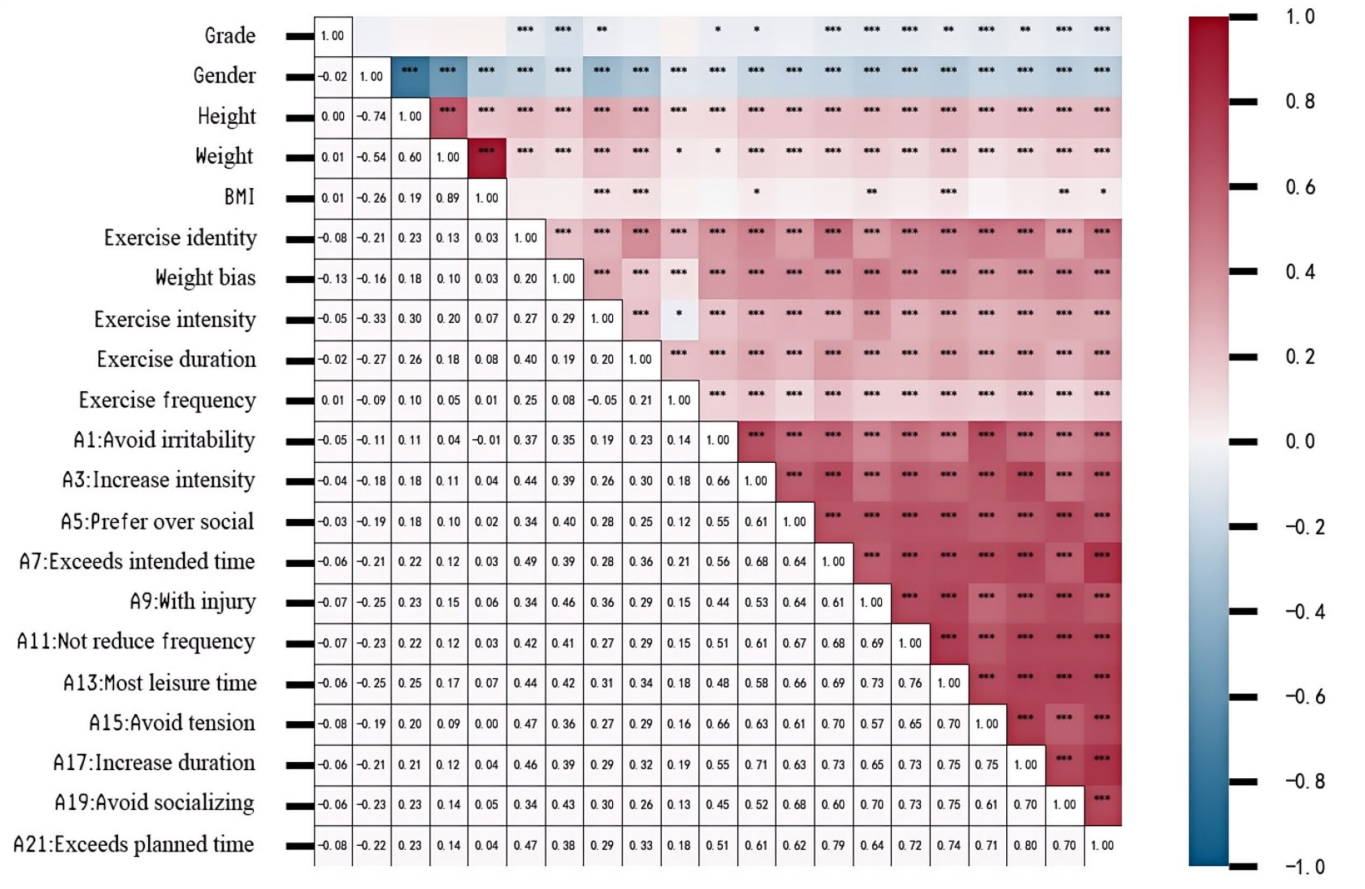
Correlation heatmap of demographic, predictor variables related to exercise dependence.

To establish the major features of exercise dependence behavior and its associated variables, we calculated correlation coefficients between the demographic factors, physical measures, psychological predictors and EDS-R scores (see Figure 3). The analysis revealed that gender exhibited a significant negative correlation with height (*r* = −0.74), weight (*r* = −0.54), and BMI (*r* = −0.51), reflecting significant sex differences in body shape and weight distribution. Moderate positive correlations were observed among exercise intensity, duration, and frequency (*r* = 0.20–0.29), suggesting a consistent activity pattern in the multidimensional characteristics of college students’ exercise behaviors. Most EDS-R items correlated strongly with one another, with several coefficients above 0.60; in particular, *A15–A17* (*r* = 0.80), *A17–A19* (*r* = 0.80), and *A15–A19* (*r* = 0.75) formed a tightly linked cluster, suggesting a well-coupled dependence structure. Exercise identity and weight bias were moderately to strongly positively associated with the core exercise dependence items (e.g., *A17*, *A13*, *A11*, *A21*; *r*-values ≈ 0.30–0.50). Overall, correlations were generally medium to high within the dependence scale (*r* = 0.60–0.70), while strong correlations (*r* > 0.75) were concentrated in a few potential core items. These findings provide evidence of the internal consistency of the ECDS and emphasize the structure beneath exercise dependence behaviors, as well as weak relationships with physiological measures. This pattern supports the approach to use machine learning to determine a relevant predictor.

### Machine learning analysis

#### Overview of model evaluation metrics

In summary, considering that we wanted an exhaustive evaluation of the predictive models, several performance metrics were used: area under the receiver operating characteristic curve (AUC), precision, recall and *F*_1_-score. AUC represents the overall ability of the model to differentiate between classes (0.5 means by chance, and 1.0 denotes perfect discrimination). Precision measures the fraction of true high-risk students among the predicted ones, while recall (sensitivity) measures how many real positives are captured from all actual positive instances. The *F*_1_-score takes the harmonic mean of precision and recall, which allows a balance in measuring model’s performance. Also, we used both ROC and precision-recall (PR) curves. ROC curves demonstrate the trade-off between sensitivity and specificity over decision thresholds, but may give an overly optimistic view in unbalanced datasets. PR curves are a useful supplement to ROC analysis, as they concentrate on the performance of the positive class, and offer a more stringent evaluation of model applicability by avoiding false short cuts through genuine negatives.

#### Analysis of AUC values: discrimination performance of algorithms

**Figure 4:**
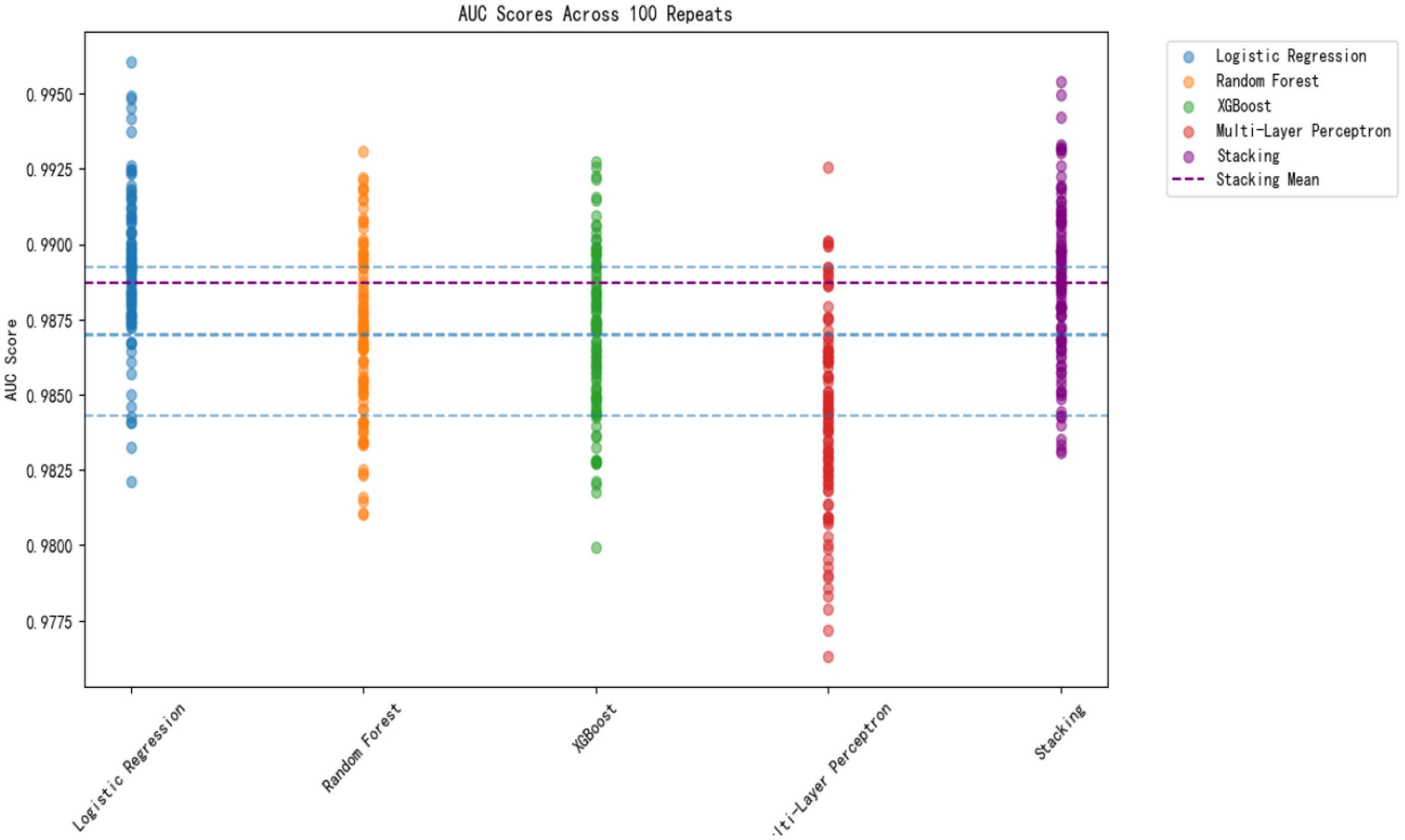
AUC scores of five machine learning models across 100 repeats.

As illustrated in Figure 4 and Table 4, all five predictive models demonstrated excellent discrimination. To rigorously evaluate the generalization ability of the proposed models, we report the performance metrics exclusively based on the independent test set using repeated (*n* = 100) hold-out validation.

**Table 4:** Performance metrics of machine learning algorithms on the independent test set.

Model	AUC ⬆	Accuracy ⬆	Precision ⬆	Recall ⬆	*F*_1_-score ⬆
Multinomial logistic regression	0.91	0.85	0.80	0.78	0.79
Random forest	0.93	0.90	0.88	0.85	0.86
XGBoost	0.94	0.89	0.87	0.84	0.85
Multilayer perceptron	0.95	0.87	0.84	0.80	0.82
Stacking learning	0.96	0.91	0.89	0.86	0.87

The stacking learning framework demonstrated superior performance, achieving a maximum mean AUC of 0.96 and an accuracy of 0.91, outperforming individual base classifiers (LM, RF, and XGBoost). While the multilayer perceptron (MLP) showed a slightly lower accuracy of 0.87 compared to the ensemble method, it still maintained a robust predictive capability (AUC = 0.95).

All models were significantly superior to chance level (50%, *p* < 0.001). The confidence intervals across Table 4 confirm that these models are not overfitting or simply memorizing the training data, but rather learning stable patterns that generalize well to novel instances. The AUC distribution further indicates that, although single models performed well, stacking learning provided the best clear separation for identifying different risk levels of exercise dependence.

#### Precision-recall performance: reliability across risk levels

**Figure 5:**
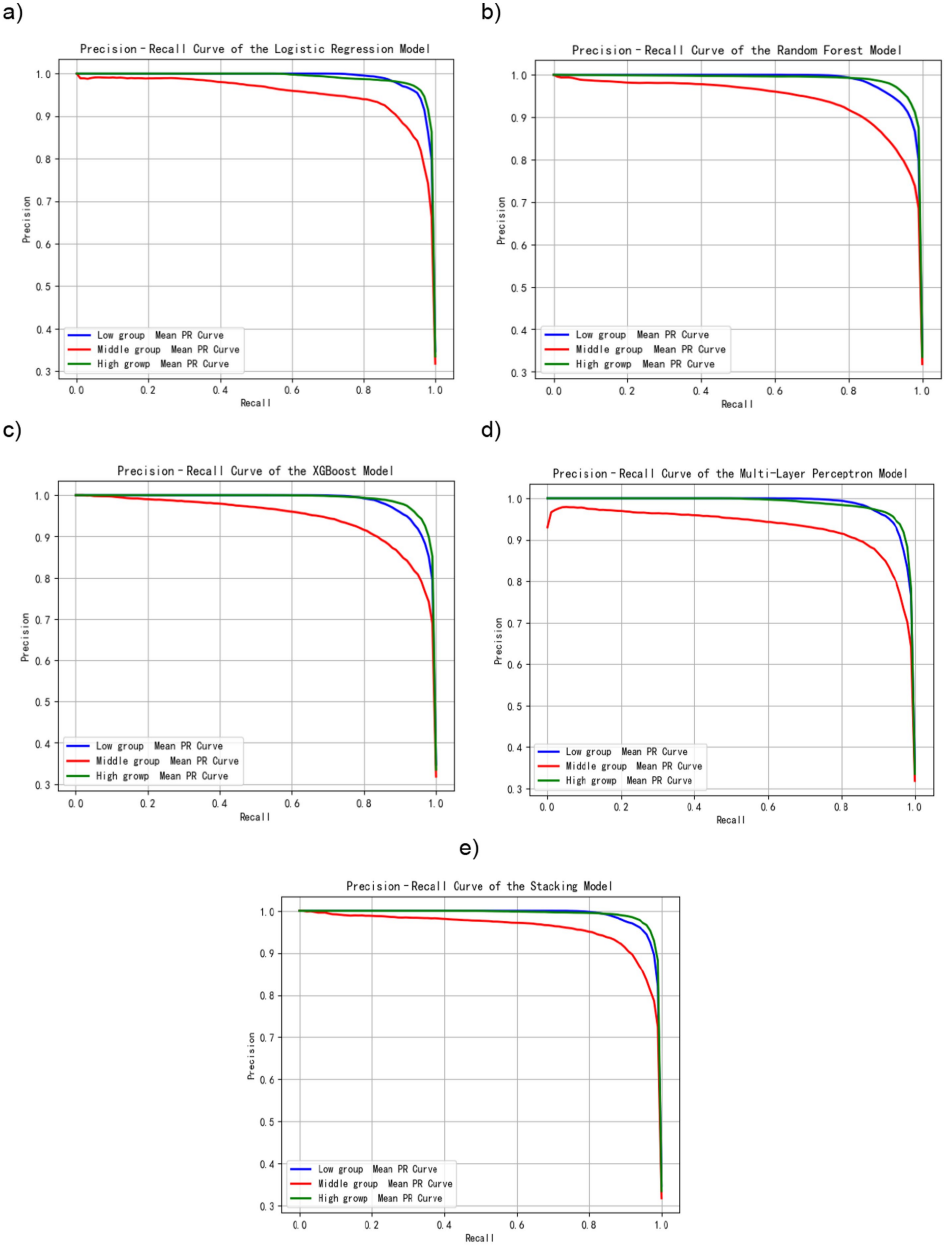
PR curves of machine learning models for exercise dependence risk. **(a)** Precision-recall curve of the logistic regression model. **(b)** Precision-recall curve of the random forest model. **(c)** Precision-recall curve of the XGBoost model. **(d)** Precision-recall curve of the multilayer perceptron model. **(e)** Precision-recall curve of the stacking model.

Figure 5 shows the precision-recall (PR) curves for the four base models and the stacking model for each of the three exercise dependence levels. Average precision was high for every model and for each risk group, with the PR curves were all pushed towards the upper-right corner. All models performed well in three risk levels, with the PR curves approaching the ideal point (1, 1). The Stacking ensemble model did better than any single model, with AP values of 0.9922, 0.9632, and 0.9928 for the low, medium, and high-risk groups, respectively. These high AP scores suggest that the models maintain high precision even at high recall levels, effectively minimizing false positives and confirming that the high accuracy is not an artifact of overfitting to the majority class.

#### Classification accuracy and confusion matrix analysis results

**Figure 6:**
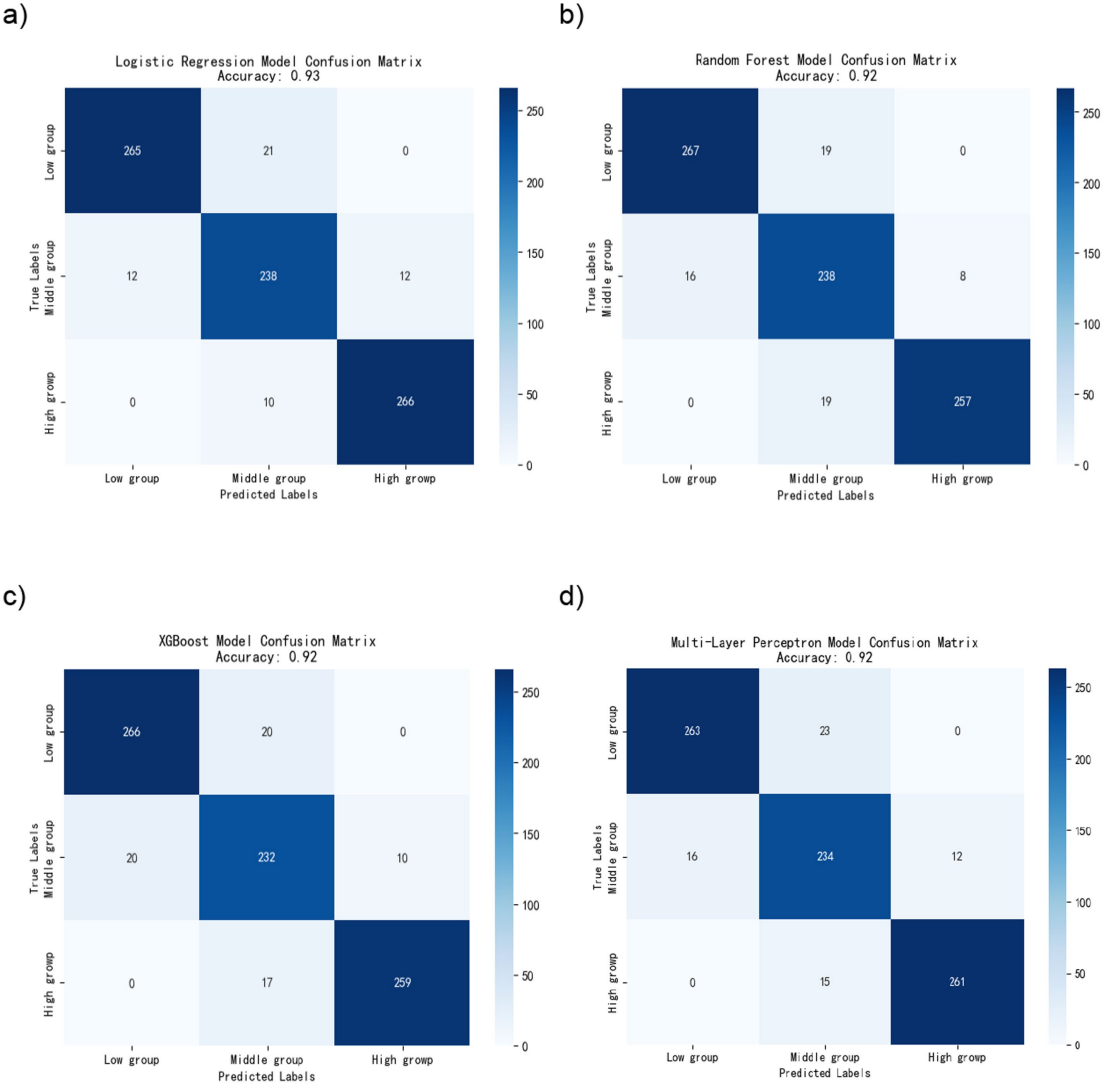
Confusion matrix diagrams for four machine learning models. **(a)** Logistic regression model. **(b)** Random forest model. **(c)** XGBoost model. **(d)** Multilayer perceptron model.

Figure 6 shows the confusion matrices for the four models. Cells on the main diagonal represent correct classification; off-diagonal cells indicate errors. All four models distinguished the three risk groups effectively. Logistic regression and the multilayer perceptron (MLP) reached the highest accuracy (accuracy = 0.98), random forest reached 0.97, and XGBoost reached 0.96. Across the three risk groups, all models classified most students correctly. MLP and logistic regression had the highest correct counts, random forest was close, and XGBoost was slightly behind.

#### Model stability and robustness verification

**Figure 7:**
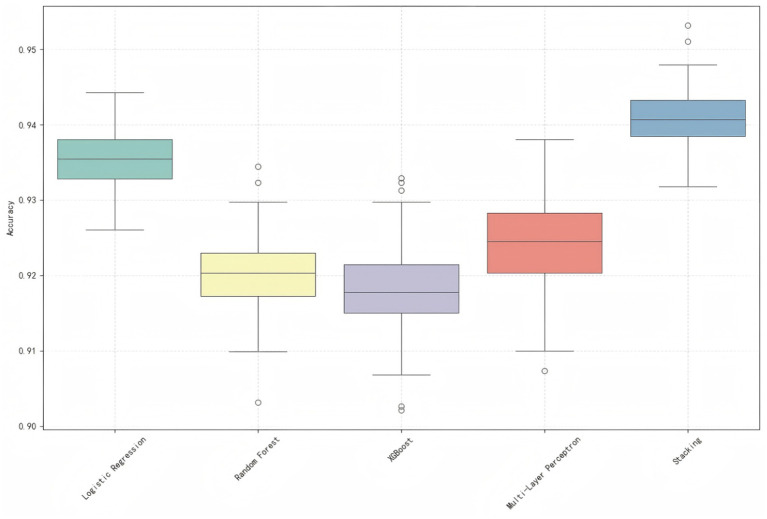
Accuracy distribution of five machine learning models over 100 repeated 5-fold cross-validations.

To alleviate the overfitting and validate model calibration, we further carried out a rigorous robustness test through 100 repetitions of 5-fold cross-validation. The accuracy distribution of four single models and stacked model is shown in Figure 7. The results indicate excellent stability. Performance of the stack is the most accurate and stable, having the highest median accuracy (≈0.94) and smaller IQR (0.93–0.95). Logistic regression had relatively low variances as well. The small deviation of accuracy over the 100 runs indicates a well-calibrated model which is robust against random splits in the training data.

In contrast, the median accuracy for random forest, XGBoost and MLP models was slightly lower (around 0.92–0.93) and somewhat more variable—in some cases down to around 0.90 but also retained high baseline performance. The stronger stability provided by the stacking model and logistic regression is consistent with their ranking in interpreting the conclusion, that high level prediction power results from systematic rather than overfitting pattern of findings.

#### Feature importance analysis in the random forest model

**Figure 8:**
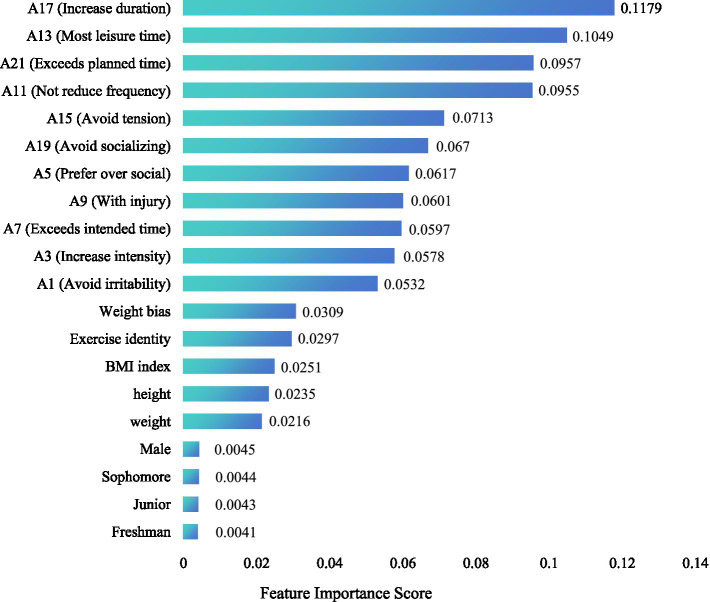
Random forest feature importance ranking (top 20).

To understand how the model has “decided” to predict the outcomes, the team performed an analysis of the feature importance of the predictors. For RF models, feature importance is commonly measured by the mean decrease in impurity approach, in which the impurity is determined by splitting a specific tree on a certain variable, and then averaged across all trees. A higher score reflects a more important role of the variable in classification according to the factor. Figure 8 presents the most important predictive variables estimated by the RF model for exercise dependence risk, encompassing the top 20 by contribution. The data in the chart reflect feature importance scores on the *x* axis and predictors under consideration on the *y* axis. The most important feature included specific indicators of exercise dependence severity. More specifically, the three most important variables were *A17* (“Increase duration,” with the score of *A17* = 0.1179), *A13* (“Most leisure time,” with the score of *A13* = 0.1049), and *A21* (“Exceeds planned time,” with the score of *A21* = 0.0957). These variables with the 3 top importance scores for the model emphasized more exercise due to tolerance and the consumption of more time to be critical for the identification of exercise dependence development. Additionally, the most important variables were *A11* (“Not reduce frequency”); *A15* (“Avoid tension”); *A19* (“Avoid socializing”); *A5* (“Prefer over social”); *A9* (“With injury”); *A7* (“Exceeds intended time”); *A3* (“Increase intensity”) and *A1* (“Avoid irritability”).

Overall, withdrawn symptoms and preferred activities over socialization show major importance for dependent behaviors. In contrast, psychological and physiological variables, such as weight bias internalization, exercise identity, and BMI, show low importance scores. However, they are crucial for maximizing the overall prediction accuracy of the model. Lastly, demographic factors such as gender and year of study show the lowest importance scores. This means that demographic components had minimal prediction power for exercise dependence in this specific group.

#### Shapley value analysis of predictors in the final model from the stacking learning method

**Figure 9:**
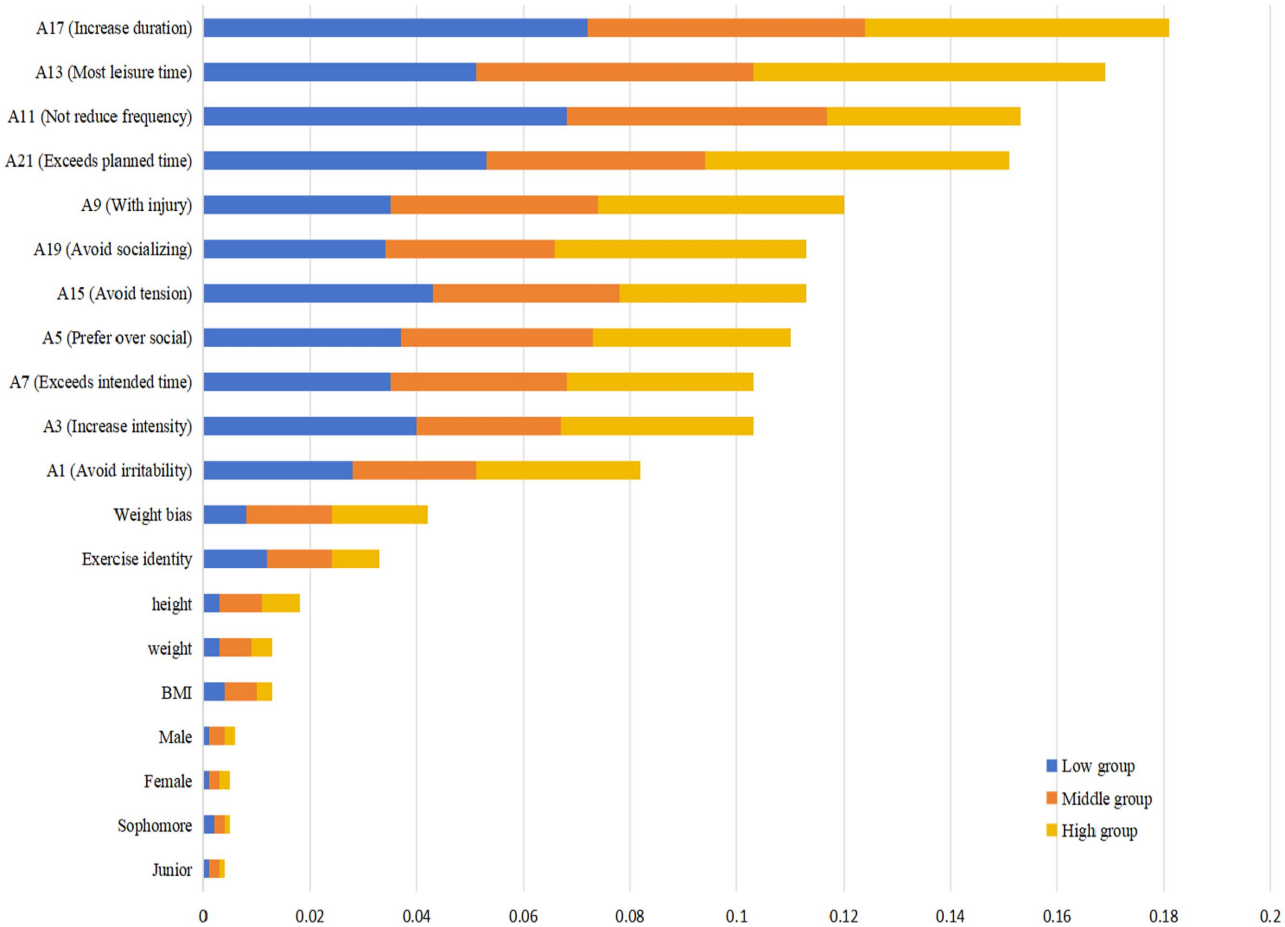
Summary of SHAP values in the random forest model.

To further examine how each predictors contributed to exercise dependence, we computed for all variables in the stacked model using the holdout set and get the SHAP values. All predictors showed SHAP values significantly above zero (*p*-values <0.05), indicating that each variable added unique information to the model’s predictions. According to the ranking of Shapley values, Table 5 and Figure 9 summarizes the SHapley Additive exPlanations (SHAP) values for key predictors whose marginal contributions to the prediction of exercise dependence risk levels were greater than or equal to 1%. The clear differences between variables indicate that some predictors played a much larger role than others in estimating exercise dependence risk in college students. The predictors could be grouped into four categories based on their importance, from highest to lowest.

**Table 5:** Mean SHAP values of predictors across exercise dependence risk groups in the stacking ensemble model.

Predictor	Junior	Sophomore	Female	Male	BMI	Weight	Height	Exercise identity	Weight bias	*A1*	*A3*	*A7*	*A5*	*A15*	*A19*	*A9*	*A21*	*A11*	*A13*	*A17*
Low-risk group SHAP value	0.001	0.002	0.001	0.001	0.004	0.003	0.003	0.012	0.008	0.028	0.040	0.035	0.037	0.043	0.034	0.035	0.053	0.068	0.051	0.072
Medium-risk group SHAP value	0.002	0.002	0.002	0.003	0.006	0.006	0.008	0.012	0.016	0.023	0.027	0.033	0.036	0.035	0.032	0.039	0.041	0.049	0.052	0.052
High-risk group SHAP value	0.001	0.001	0.002	0.002	0.003	0.004	0.007	0.009	0.018	0.031	0.036	0.035	0.037	0.035	0.047	0.046	0.057	0.036	0.066	0.057
Mean SHAP value (%)	0.004	0.005	0.005	0.006	0.013	0.013	0.018	0.033	0.042	0.082	0.103	0.103	0.110	0.113	0.113	0.120	0.151	0.153	0.169	0.181

The first group is the core contributing variables (Shapley values ≥5%), including *A17*, *A13*, *A11*, and *A21*, with Shapley values significantly higher than other variables, reaching approximately 17.5, 16, 15, and 14%, respectively. In the low-risk group, items *A17* (0.072), *A11* (0.068), *A21* (0.053), and *A13* (0.051) showed noticeably higher SHAP values than the remaining predictors, indicating that they played the main role in the model for this group. The same items stayed at the top in the medium- and high-risk groups (mean SHAP ≈ 0.041–0.066), which supports their status as core indicators. In the high-risk group, for example, *A13* and *A21* reached 0.066 and 0.057, respectively, well above most other variables. Paired-sample t tests did not detect significant differences in the average SHAP values of *A17* and *A21* across groups (*t* = 1.82, df = 2, *p* > 0.05), but both items remained consistently higher than the rest, confirming their central contribution to distinguishing exercise-dependence levels in this sample.

The second group of variables had Shapley values between 2 and 4% and included *A9*, *A19*, *A15*, *A5*, *A7*, *A3* and *A1*. These made a moderate contribution, with *A19* and *A9* standing out in the high-risk group (0.047 and 0.046), close to or slightly above 4%. Their values fluctuated less than those of the core items and form a clear step below the first tier. This middle level showed fairly even predictive strength, which made the model’s importance structure appear graded.

The third group of variables had Shapley values between about 0.01 and 0.02, consisted mainly of the total scores for exercise identity and weight bias. In the high-risk group, their SHAP values were 0.018 and 0.009, and although slightly lower in the other groups, they remained above 0.01. Thus, while weaker than the first two tiers, these variables still added explanatory value to the model. These results indicate that although exercise identity and weight bias totals were weaker than the first two tiers, they still added useful explanatory power to the model.

The fourth group consisted of variables with low marginal contributions (SHAP <1%), primarily demographic and basic physical measurements such as height, weight, BMI, gender, and academic year. For example, height and weight had SHAP values below 0.01 in every group, and BMI, gender, and year of study were even lower. Although these variables reached statistical significance, their marginal contribution to the model was small. This suggests that college students’ primary predictors of exercise dependence behavior lie in psychological and behavioral factors rather than in basic demographic or physical measurements.

The Shapley value analysis results in this study demonstrate that the key predictors of exercise dependence behavior among college students are mainly concentrated in the core variables *A17* (Increase duration), *A13* (Most leisure time), *A11* (Not reduce frequency), and *A21* (Exceeds planned time). These variables not only align closely with the core pathological features of exercise dependence, but also exhibit dominant contributions in the Stacking ensemble model. Additionally, scale-based variables such as the total scores for exercise identity and weight bias, along with certain behavioral indicators, provided auxiliary support to the model’s predictive power. In contrast, demographic and physical measurement indicators (e.g., height, weight and BMI) played a relatively limited role.

## Discussion

### Prevalence, group differences, and ML-based identification of key predictors among college students

The present study was motivated by two gaps in the literature: (a) exercise dependence has rarely been examined in Chinese university contexts from a theory-driven perspective, and (b) applications of deviance regulation theory (DRT) have seldom been combined with machine-learning models capable of capturing non-linear identity processes. Our findings address both gaps by linking SHAP-derived importance patterns to core DRT constructs of identity regulation and norm-contingent motivation. This study seeks to provide a characterization of how prevalent exercise dependence is in a large Chinese college student sample, across basic demographic categories, and which psychological/behavioral identifiers are most useful for demarcating type patterns. Drawing on high volume survey data and machine learning techniques (Pedregosa et al., 2011), we provided important insights into the complex relationship patterns between psychological and demographic factors from DRT. The main findings are presented in the following below.

First, the present study revealed that some 33% of university students are potentialy exposed to exercise dependence which approximate similar results between domestic and foreign literature (Allegre et al., 2006; Garman et al., 2004; Živkov et al., 2022). The data imply that when the frequencies and intensities of exercising become elevated, a sub-group of students cross from healthy involvement to unhealthy dependence, which is marked by compulsive control and inflexible routines. In the absence of effective self-regulation, or environmental cues to reduce these behaviors, they become established, and early identification is essential. This result reflects the widespread but unreported phenomenon of exercise dependence in universities and confirms earlier findings about increasing prevalence of exercise addiction (Berczik et al., 2014; Symons Downs et al., 2019). Moreover, we obtained better classification performance as compared to traditional psychometric cut-offs by using an ensemble of machine learning models (logistic, random forest, tree-based XGBoost and MLP). The ensemble learning model was the best predictor (mean AUC = 99.16%). The model had excellent predictive discrimination when the AUC was converted to Cohen’s *d* effect sizes (>2.0). Unlike the previous studies that have used only linear statistical techniques, our method also demonstrates that data-driven models can successfully disentangle non-linear aspects of exercise behaviors and can be used as a powerful instrument to distinguish high risk individuals from general population (Goh et al., 2022; Gulhane and Sajana, 2021).

Second, demographic profiling revealed that male students, freshmen and sophomores, and those with normal BMI exhibited significantly higher exercise dependence scores compared to their counterparts. These results corroborate Garman’s et al. (2004) observation that the early university years represent a critical window of vulnerability for developing exercise dependence. According to correlation analyses, dependence was moderately related to psychological variables—partially with exercise identity and weight cognition bias—but not with physiological measurements (e.g., BMI). This is consistent with the interpretation that EDS reflects mainly psychological reinforcement and habit formation (Hausenblas and Symons Downs, 2002) At least from DRT perspective, however, these demographic and psychological trends imply those students could be at risk for perceiving their exercise behavior as a necessary tool to control perceived self-discrepancies.

Third, Shapley value analysis disentangled the relative importance of specific predictors, revealing that tolerance and time-allocation behaviors—specifically items *A17* (Increasing duration), *A13* (Spending leisure time on exercise), *A11* (Inability to reduce frequency), and *A21* (Exercising longer than planned)—were the paramount predictors, with contributions ranging from 14 to 17.5%. These findings strongly implicate loss of control and excessive time investment as the core pathology of the disorder, consistent with previous literature (Allegre et al., 2006; Symons Downs et al., 2019). Critically, the prominence of *A17* accords with predictions based on deviation regulation theory. When people notice that they do not meet an attractive standard (e.g., physical fitness, body image availability), in the DRT model, they enhance whatever behavior differs distinctly most from what is believed to be necessary so that they will fit this standard or return to it (Blanton and Christie, 2003). In the context of exercise dependence, this manifests as a maladaptive feedback loop: to eliminate cognitive dissonance regarding body image or performance, individuals escalate their investment, leading to the observed “tolerance” effect. In DRT terms, these patterns imply that students escalate exercise precisely because they perceive themselves as deviating from an internalized ideal, thereby creating a norm-contingent feedback loop in which identity threats are managed through increasingly rigid exercise routines. The high Shapley values for *A13*, *A11*, and *A21* further illustrate the collapse of self-regulatory systems, where the motivation to exercise overrides other life priorities (Köpetz et al., 2013). Secondary predictors (e.g., *A9*, *A19*; contribution 2–4%) and tertiary psychological factors (e.g., exercise identity; contribution 1–2%) provided supplementary predictive power. One potential hierarchical model of this nature is such that overarching psychological forces lay the groundwork, but it is students’ observable behaviors regarding a “loss of control” that are the only culturally sanctioned markers of at-risk status.

On the other hand, in contrast to the biases of psychological factor preferences discussed above, basic demographic and physiological measurement-related factors held consistent below 1% Shapley values. This discovery contributes a particularly important theoretical insight in that it implies the constructs of exercise dependence are not intrinsic to certain demographic profiles, but rather reflective of cognitive self-regulatory processes. More specifically, these findings expand upon DRT as a theoretical framework and specify the process whereby “identity regulation” and “stigma avoidance” work. DRT postulates that people conform on norms to develop a positive self concept, or avoid the “stigma of nonconformity. That psychological, but not demographic, predictors had relatively high feature importance suggests exercise dependence represents a compensatory phenomenon. Excessive exercise among students probably has less to do with biological factors (e.g., the BMI) than maintaining identity in the face of perceived social norms, namely warding off stigma internalized from being seen as “lazy” or “unfit.” This reading goes beyond descriptive validation, and suggests a behavioral dependence that is, at its core, a paradoxical maladaptive byproduct of successful identity management.

This trend is also consistent with recent machine learning work on behavioral addictions such as Hong et al. (2024) about smartphone addiction in which self-regulation and motivational control play a key role inherent to more static traits. But it contrasts with the results of Ferrer et al. (2011) and Dvorak et al. (2016, 2018) related to substance use disorder, while the focus in these studies was mainly on socio-demographic risk factors. Our finding that the distinctive psychological dominancy (exercise dependence) it represents contributes to a literature review in sports psychology, adding information rather than demographic vulnerability, what is specifically for exercise dependence in particular—norm-contingent motivation.

While the direct effects of demographic factors are unimpressive; yet, their subtle possible roles should be taken into account. For example, gender or grade may not be an overarching determinant of dependence, but rather act as a moderating factor on the strength with which psychological pressures affect individuals (e.g., weight stigma effects may differ by gender). Tentative forays demonstrated that the role of core predictors such as *A17* was slightly more pronounced in males and first-year students, indicating possible moderator effects. Future studies may use multi-level linear models to better disentangle these associations.

### Innovative use of machine-learning modeling and interpretation

Here, we combined ensemble machine-learning models to break down exercise dependence. As noted by Bartlett et al. (2014), these models suggest enhanced exclusive tracking of behavioral data via non-linear content that traditional linear counterparts might miss: importantly and uniquely, our SHAP translation of “black-box” algorithms allowed us to create theoretically interpretable outputs and trace a prediction back to distinct “norm-deviation” behaviors and identity indicators. This interpretability serves as the bridge between advanced data science and actionable intervention planning. This insight allows for the operationalization of findings into two distinct practical avenues. First, in university counseling settings, the high-ranking item-level predictors can be utilized to develop rapid screening protocols. Instead of administering full-length diagnostic batteries, counselors can prioritize these “red flag” questions to efficiently triage students at risk (Lichtenstein et al., 2014b). Second, in athletic settings, these insights enable coaches to distinguish between high-performance training and pathological dependence. By monitoring specifically identified behaviors—such as the inability to reduce exercise intensity despite injury—staff can implement targeted, symptom-specific interventions rather than generic exercise reduction strategies, thereby preventing the escalation of dependence (Nogueira et al., 2018; Szabo et al., 2015).

### Limitations and future directions

This study has some limitations, although it makes a significant contribution. First of all, the model did not involve variables such as social support or peer norms so some variance in DRT’s “social context” component was unaccounted for. Second, self-report questionnaires may be subject to reporting biases. Third, the sample is not regionally diverse. Studies combining objective physiological assessments, inclusion of multi center populations and incorporating data from wearable technology to capture identity regulation through exercise *in vivo* are also warranted.

## Conclusion

To the best of our knowledge, for the first time various artificial intelligence models are used to investigate exercise dependence in Chinese college students. Using data on 2,745 students, we examined prevalence trends and demographic correlates as well as the main predictors. 33% of the students exhibited risk signals, whereas males and lower class levels and normal BMI students had a higher proportion of risk. The AUC of the 4-classifier-based stacked model was very high (99.16%), allowing accurate risk stratification. The model-interpretation results demonstrated that for prediction, behavioral/psychological items were more informative than demographics. There are 4 of them: extending exercise time to achieve the desired effect (*A17*), spending maximum time with leisure activity-exercising way (*A13*), not able to reduce frequency (*A11*) and exercising longer than plan script *A21*. These measures are directly related to excessive control and excess investment. They are also congruent with the compensatory regulation theory of exercise dependence.

The study, in general, highlights the promise of artificial intelligence towards capacity-building within sport and exercise psychology by providing new methods for detecting risk and developing evidence-informed intervention. It would be a valuable task for the future to refine current models, validate them in other university settings, and integrate them with more realistic intervention scenarios aimed at enhancing healthy exercise behaviors in college students.

## Data Availability

The original contributions presented in the study are included in the article/Supplementary material, further inquiries can be directed to the corresponding author.
