# Correction: Predicting college students' exercise dependence: a machine learning approach

**DOI:** 10.3389/fpsyg.2026.1892436

**Published:** 2026-07-09

**Authors:** Yihang Deng, Wei Lan, Mingda Si, Yi Lin Ren

**Affiliations:** 1 Department of Physical Education, Neijiang Normal University, Neijiang, China; 2 Embodied Media Laboratory, Graduate School of Media Design, Keio University, Hiyoshi Campus, Yokohama, Japan; 3 National Institute of Education, Nanyang Technological University, Singapore, Singapore; 4 Zhuhai Research Center for Women and Children's Sports Culture, College of Sports, Jinan University Zhuhai Campus, Zhuhai, Guangdong, China

**Keywords:** college student, ensemble learning (EN), exercise dependence behavior, machine learning, risk prediction

Equations in the section “**Methods**,” subsection “*Data analysis*,” under “*Four machine learning algorithms and procedure*s” and “*Evaluation metrics*,” were erroneously given as:

Accuracy = (TP + TN + FP + FN) / (TP + TN)

Precision = (TP + FP) / TP

Recall = (TP + FN) / TP

The correct equations are:

Accuracy = (TP + TN)/(TP + TN + FP + FN)

Precision = TP/(TP + FP)

Recall = TP/(TP + FN)

The original version of this article has been updated.

