# Supplementary material for: Predicting college students’ exercise dependence: a machine learning approach
Source: Front Psychol. 2026 Jan 29;17:1743725. doi: 10.3389/fpsyg.2026.1743725 (PMC12895345; doi:10.3389/fpsyg.2026.1743725)
Supplement: Supplementary file 1 [file Supplementary_file_1.docx]

**Appendix**

**Appendix A: Machine Learning Algorithm Settings**

This section lists the full hyperparameter and model settings used for the four machine-learning algorithms (multinomial logistic regression, random forest, XGBoost, and multilayer perceptron), including the following:

Multinomial Logistic Regression (MLR)

- Regularization: L2 regularization (penalty='l2')
- Solver: lbfgs (for medium-sized datasets)
- Maximum Iterations: 1000
- Regularization Strength: C = 1.0

Random Forest (RF)

- Number of Trees: n_estimators = 100
- Maximum Depth: None
- Criterion: Gini index
- Minimum Samples Split: 2

XGBoost

- Number of Trees: 100
- Learning Rate: 0.3
- Maximum Tree Depth: 6
- Evaluation Metric: Log loss

Multilayer Perceptron (MLP)

- Hidden Layers: 64 and 32 neurons
- Activation Function: ReLU
- Optimizer: Adam
- Learning Rate: 0.001
- L2 Regularization: 0.0001

**Appendix B: Detailed Model Training and Testing Procedure**

This section describes how the models were built and evaluated, including data splitting, feature processing, and use of SMOTE (Synthetic Minority Over-sampling Technique). It also reports the main performance indices (accuracy, precision, recall, F1) for each model in table form.

Model Training and Testing Procedure:

1. Dataset Partitioning:
   - The dataset is divided into a training set and a testing set, with the training set typically comprising 70%-80% of the total data, and the remaining portion used as the testing set.
   - Five-fold cross-validation is employed to ensure robust performance evaluation.
2. Feature Engineering:
   - Data preprocessing steps include handling missing values, encoding categorical variables, and performing feature scaling.
   - Feature selection is carried out to remove redundant or irrelevant features, enhancing model efficiency and performance.
3. SMOTE Application:
   - SMOTE is applied to oversample the minority class in the training set, addressing any class imbalance issues and ensuring better model generalization.
4. Training and Evaluation:
   - The models are trained using standard procedures, with hyperparameters tuned for optimal performance.
   - Each model's performance is assessed, and accuracy, precision, recall, and F1-score metrics are recorded for comparison.

| Model Performance Metrics | | | | |
| --- | --- | --- | --- | --- |
| Model | Accuracy ⬆ | Precision ⬆ | Recall ⬆ | F1-score ⬆ |
| Multinomial Logistic Regression | 0.85 | 0.80 | 0.78 | 0.79 |
| Random Forest | 0.90 | 0.88 | 0.85 | 0.86 |
| XGBoost | 0.89 | 0.87 | 0.84 | 0.85 |
| Multilayer Perceptron | 0.87 | 0.84 | 0.80 | 0.82 |

**Appendix C: Stacking Method Details**

This section provides a detailed explanation of the stacking method employed, including the following components:

Base Models:

The stacking method utilizes four base models:

1. Multinomial Logistic Regression (MLR)
2. Random Forest (RF)
3. XGBoost
4. Multilayer Perceptron (MLP)

Five-Fold Cross-Validation:

- A five-fold cross-validation process is used to evaluate the performance of each base model. The dataset is divided into five subsets, with four subsets used for training and the remaining subset used for testing. This process is repeated five times to ensure comprehensive evaluation and prevent overfitting.

Logistic Regression as the Meta-Learner:

- Logistic regression is employed as the meta-learner in the stacking method. The base model predictions are combined and input into the logistic regression model to generate the final prediction.

Holdout Set Validation:

- A holdout set is used for additional validation of the stacked model, ensuring that the model's generalization ability is not compromised by data leakage during training.

Shapley Values Interpretation:

- Shapley values are employed to interpret the feature importance of each model. Shapley values, derived from cooperative game theory, provide a way to quantify each feature's contribution to the model's output, thereby enhancing model interpretability.

**Appendix D: Details of Measurement Instruments**

**1.** **Exercise Dependence Scale-Revised (EDS-R)**

Instructions: Using the scale provided below, please answer the following questions as honestly as possible. This question refers to your current exercise beliefs and behaviors that have occurred within the past 3 months. Write your answers in the space provided after each statement. 1 indicates never, 6 indicates always, with increasing frequency from 1 to 6.

| Item | 1  Never | 2 | 3 | 4 | 5 | 6  Always |
| --- | --- | --- | --- | --- | --- | --- |
| 1. I exercise to avoid feeling irritable. | 1 | 2 | 3 | 4 | 5 | 6 |
| 2. I exercise despite recurring physical problems. | 1 | 2 | 3 | 4 | 5 | 6 |
| 3. I continually increase my exercise intensity to achieve the desired effects/benefits. | 1 | 2 | 3 | 4 | 5 | 6 |
| 4. I am unable to reduce how long I exercise. | 1 | 2 | 3 | 4 | 5 | 6 |
| 5. I would rather exercise than spend time with family/friends. | 1 | 2 | 3 | 4 | 5 | 6 |
| 6. I spend a lot of time exercising. | 1 | 2 | 3 | 4 | 5 | 6 |
| 7. I exercise longer than I intend. | 1 | 2 | 3 | 4 | 5 | 6 |
| 8. I exercise to avoid feeling anxious. | 1 | 2 | 3 | 4 | 5 | 6 |
| 9. I exercise when injured. | 1 | 2 | 3 | 4 | 5 | 6 |
| 10. I continually increase my exercise frequency to achieve the desired effects/benefits. | 1 | 2 | 3 | 4 | 5 | 6 |
| 11. I am unable to reduce how often I exercise. | 1 | 2 | 3 | 4 | 5 | 6 |
| 12. I think about exercise when I should be concentrating on school/work. | 1 | 2 | 3 | 4 | 5 | 6 |
| 13. I spend most of my free time exercising. | 1 | 2 | 3 | 4 | 5 | 6 |
| 14. I exercise longer than I expect. | 1 | 2 | 3 | 4 | 5 | 6 |
| 15. I exercise to avoid feeling tense. | 1 | 2 | 3 | 4 | 5 | 6 |
| 16. I exercise despite persistent physical problems. | 1 | 2 | 3 | 4 | 5 | 6 |
| 17. I continually increase my exercise duration to achieve the desired effects/benefits. | 1 | 2 | 3 | 4 | 5 | 6 |
| 18. I am unable to reduce how intensely I exercise. | 1 | 2 | 3 | 4 | 5 | 6 |
| 19. I choose to exercise so that I can get out of spending time with family/friends. | 1 | 2 | 3 | 4 | 5 | 6 |
| 20. A great deal of my time is spent exercising. | 1 | 2 | 3 | 4 | 5 | 6 |
| 21. I exercise longer than I plan. | 1 | 2 | 3 | 4 | 5 | 6 |

The scoring for each dimension of this scale is as follows: Continuance: items 2, 9, 16; Tolerance: items 3, 10, 17; Lack of control: items 4, 11, 18; Reduction in other activities: items 5, 12, 19; Time: items 6, 13, 20; Intention effect: items 7, 14, 21.

**2. Exercise Identity Scale (EIS)**

Instructions: Below are several statements. Please indicate your level of agreement or disagreement by selecting one of the seven options provided after each statement and marking the corresponding number with a checkmark. (1 point = Strongly disagree, 7 points = Strongly agree.)

| Item Item | Strongly disagree |  |  |  |  |  | Strongly agree |
| --- | --- | --- | --- | --- | --- | --- | --- |
| 1. I consider myself an exerciser. | 1 | 2 | 3 | 4 | 5 | 6 | 7 |
| 2. When introducing myself to others, I mention that I exercise. | 1 | 2 | 3 | 4 | 5 | 6 | 7 |
| 3. I have many exercise-related goals. | 1 | 2 | 3 | 4 | 5 | 6 | 7 |
| 4. Exercise is very important to me. | 1 | 2 | 3 | 4 | 5 | 6 | 7 |
| 5. I need exercise to feel better. | 1 | 2 | 3 | 4 | 5 | 6 | 7 |
| 6. Others see me as someone who exercises regularly. | 1 | 2 | 3 | 4 | 5 | 6 | 7 |
| 7. For me, being an exerciser means more than just doing exercise. | 1 | 2 | 3 | 4 | 5 | 6 | 7 |
| 8. I feel it's a big loss when I can't exercise. | 1 | 2 | 3 | 4 | 5 | 6 | 7 |
| 9. I constantly think about exercise. | 1 | 2 | 3 | 4 | 5 | 6 | 7 |

**3. Eating Pathology Symptoms Inventory (ESPI)**

Note: The following five sets of statements describe common experiences or issues encountered by ordinary people in daily life. Please read them carefully and select the option that best reflects your behavior over the past four weeks (including today). Answer using the following options: 1—Never 2—Rarely 3—Sometimes 4—Often 5—Always. Mark the corresponding number with a checkmark (√).

| Item | Never | Rarely | Sometimes | Often | Always |
| --- | --- | --- | --- | --- | --- |
| 1. Seeing fat people makes me feel sick. | 1 | 2 | 3 | 4 | 5 |
| 2. I think overweight people are lazy. | 1 | 2 | 3 | 4 | 5 |
| 3. I think obese people lack self-control. | 1 | 2 | 3 | 4 | 5 |
| 4. I think overweight people are unattractive. | 1 | 2 | 3 | 4 | 5 |
| 5. Seeing fat people in tight clothes makes me feel sick. | 1 | 2 | 3 | 4 | 5 |
